# Concepts and Outcomes of Perioperative Therapy in Stage IA-III Pancreatic Cancer—A Cross-Validation of the National Cancer Database (NCDB) and the German Cancer Registry Group of the Society of German Tumor Centers (GCRG/ADT)

**DOI:** 10.3390/cancers14040868

**Published:** 2022-02-09

**Authors:** Louisa Bolm, Sergii Zemskov, Maria Zeller, Taisuke Baba, Jorge Roldan, Jon M. Harrison, Natalie Petruch, Hiroki Sato, Ekaterina Petrova, Hryhoriy Lapshyn, Ruediger Braun, Kim C. Honselmann, Richard Hummel, Oleksii Dronov, Alexander V. Kirichenko, Monika Klinkhammer-Schalke, Kees Kleihues-van Tol, Sylke R. Zeissig, Dirk Rades, Tobias Keck, Carlos Fernandez-del Castillo, Ulrich F. Wellner, Rodney E. Wegner

**Affiliations:** 1 Department of Surgery, Massachusetts General Hospital and Harvard Medical School, Boston, MA 02114, USA; tbaba1@mgh.harvard.edu (T.B.); roldan.md@hotmail.com (J.R.); jmharrison@partners.org (J.M.H.); natalie@petruch.com (N.P.); hirokisato@asahikawa-med.ac.jp (H.S.); cfernandez@mgh.harvard.edu (C.F.-d.C.); 2 Department of Surgery, University Medical Center Schleswig-Holstein, Campus Luebeck, 23562 Luebeck, Germany; m.zeller92@web.de (M.Z.); ek.petrova@yahoo.de (E.P.); hryhoriy.lapshyn@uksh.de (H.L.); ruediger.braun@uksh.de (R.B.); kim.honselmann@uksh.de (K.C.H.); richard.hummel@uksh.de (R.H.); tobias.keck@uksh.de (T.K.); ulrich.wellner@uksh.de (U.F.W.); 3 Department of General Surgery, Bogomolets National Medical Unoversity, 01601 Kyiv, Ukraine; szemskovmeister@gmail.com (S.Z.); ai_dronov@ukr.net (O.D.); 4 Division of Radiation Oncology, Allegheny Health Network Cancer Institute, Pittsburgh, PA 15224, USA; alexander.kirichenko@ahn.org (A.V.K.); rodney.wegner@ahn.org (R.E.W.); 5 German Cancer Registry Group, Society of German Tumor Centers—Network for Care, Quality and Research in Oncology, 14057 Berlin, Germany; monika.klinkhammer-schalke@klinik.uni-regensburg.de (M.K.-S.); kleihuesvantol@adt-netzwerk.de (K.K.-v.T.); 6 Institute for Clinical Epidemiology and Biometry, University of Wuerzburg, 97070 Wuerzburg, Germany; zeissig@krebsregister-rlp.de; 7 Department of Radiation Oncology, University Medical Center Schleswig-Holstein, Campus Luebeck, 23538 Luebeck, Germany; dirk.rades@uksh.de

**Keywords:** pancreatic cancer, perioperative therapy, neoadjuvant therapy, pancreatic surgery

## Abstract

**Simple Summary:**

The aim of this study is to assess perioperative therapy in stage IA-III pancreatic cancer cross-validating the German Cancer Registry Group of the Society of German Tumor Centers—Network for Care, Quality, and Research in Oncology, Berlin (GCRG/ADT) and the National Cancer Database (NCDB). The cross-validation of both registries demonstrated that strategies of perioperative therapy remain consistent across the registries for stage IA-III pancreatic cancer. Combined neoadjuvant and adjuvant therapy improved overall survival as compared to either therapy alone.

**Abstract:**

(1) Background: The aim of this study is to assess perioperative therapy in stage IA-III pancreatic cancer cross-validating the German Cancer Registry Group of the Society of German Tumor Centers—Network for Care, Quality, and Research in Oncology, Berlin (GCRG/ADT) and the National Cancer Database (NCDB). (2) Methods: Patients with clinical stage IA-III PDAC undergoing surgery alone (OP), neoadjuvant therapy (TX) + surgery (neo + OP), surgery+adjuvantTX (OP + adj) and neoadjuvantTX + surgery + adjuvantTX (neo + OP + adj) were identified. Baseline characteristics, histopathological parameters, and overall survival (OS) were evaluated. (3) Results: 1392 patients from the GCRG/ADT and 29,081 patients from the NCDB were included. Patient selection and strategies of perioperative therapy remained consistent across the registries for stage IA-III pancreatic cancer. Combined neo + OP + adj was associated with prolonged OS as compared to neo + OP alone (17.8 m vs. 21.3 m, *p* = 0.012) across all stages in the GCRG/ADT registry. Similarly, OS with neo + OP + adj was improved as compared to neo + OP in the NCDB registry (26.4 m vs. 35.4 m, *p* < 0.001). (4) Conclusion: The cross-validation study demonstrated similar concepts and patient selection criteria of perioperative therapy across clinical stages of PDAC. Neoadjuvant therapy combined with adjuvant therapy is associated with improved overall survival as compared to either therapy alone.

## 1. Introduction

Pancreatic ductal adenocarcinoma (PDAC) is associated with a dismal prognosis, and early local and systemic tumor spread [1,2]. Complete oncologic resection remains the only curative option in PDAC patients, and a minority of patients diagnosed with PDAC present with initially resectable tumors [3,4]. The role of perioperative therapy has become more important, and neoadjuvant therapy is more and more performed in PDAC [5,6]. Neoadjuvant treatment may result in the downstaging, potentially leading to a higher rate of complete resections and improvement of overall survival [7].

An increasing number of national cancer registries have been established over the past years. These databases may serve to assure quality control and to evaluate multiple dimensions of patient outcomes [8,9]. National cancer registries such as the U.S.-American National Cancer Database (NCDB) and the German Cancer Registry Group of the Society of German Tumor Centers—Network for Care, Quality, and Research in Oncology (ADT), Berlin (GCRG/ADT) cover a vast portion of the nationwide caseload of cancer patients, which also applies to those diagnosed with PDAC. Comparisons of national cancer registries allow for large-scale cross-validations of treatment effects. In PDAC patients, the optimal sequence of perioperative therapy remains unclear. To date, several obstacles of perioperative therapy for PDAC need to be addressed. Patient selection criteria and perioperative concepts, and treatment strategies have not been investigated in a population-based approach on an international level. It is unclear whether neoadjuvant therapy is beneficial in all stages of potentially resectable PDAC patients and whether neoadjuvant radiochemotherapy improves prognosis when compared to neoadjuvant chemotherapy alone. Furthermore, the effect of adjuvant therapy following neoadjuvant therapy has not been determined yet. We aimed to perform a cross-validation of the U.S.-American National Cancer Database (NCDB) and the German Cancer Registry Group of the Society of German Tumor Centers—Network for Care, Quality, and Research in Oncology (ADT), Berlin (GCRG/ADT) to evaluate national standards of perioperative therapy and long-term outcomes of perioperative neoadjuvant and adjuvant therapy regimens in patients with clinical stage IA-III PDAC.

## 2. Materials and Methods

### 2.1. Study Population

The U.S.-American National Cancer Database (NCDB) supported by the American College of Surgeons and the Commission on Cancer and the German Cancer Registry Group of the Society of German Tumor Centers—Network for Care, Quality, and Research in Oncology (ADT), Berlin (GCRG/ADT) were searched for patients with histologically confirmed PDAC. Patient data were de-identified. Ethics approval for the study was obtained from the ethics committee of the University of Luebeck (#20-319). The study period was 2000–2018 for the GCRG/ADT data set and 2004–2018 for the NCDB cohort. Patient selection for this study was performed according to the consort statement and flow diagram [10]. Patients with clinical stage IA-III were included in the study population. Exclusion criteria were no data on clinical stage, clinical stage IV, no oncological resection, missing follow-up data, and missing data on the timing of perioperative therapy.

### 2.2. Study Parameters

The following patient baseline parameters were included for the analyses: age, sex, Eastern Cooperative Oncology Group (ECOG) performance status for the GCRG/ADT registry, and Charlson-Deyo co-morbidity index for the NCDB [11]. Histopathological parameters included T stage, N stage, and R status. R status was dichotomized as R0 versus R+ according to the AJCC/UICC 7th edition [12]. For both registries, perioperative regimens included surgery alone (OP alone), neoadjuvant therapy and surgery (neo + OP), surgery and adjuvant therapy (OP + adj), and neoadjuvant therapy and surgery and adjuvant therapy (neo + OP + adj). Patients undergoing neoadjuvant therapy were dichotomized as having received radiochemotherapy and surgery (neoRCTX + OP) versus chemotherapy alone and surgery (neoCTX + OP). Perioperative treatment regimens were further dichotomized as single-agent chemotherapy versus multi-agent chemotherapy. Of note, the NCDB does not include details on the specific chemotherapy agents used or number of cycles delivered. Overall survival was defined as the time from initial diagnosis to death of the patient.

### 2.3. Statistics

For statistical analysis, IBM SPSS Statistics for Windows, Version 25.0 was used. Continuous and categorical variables were expressed as median/range and absolute/relative frequencies, respectively. To compare age, sex, ECOG performance status, and Charlson-Deyo co-morbidity index between perioperative treatment groups, chi-square testing was performed. To compare T category, N category, and R status between patients with neoadjuvant versus no neoadjuvant therapy, chi-square testing was used as well. For head-to-head comparisons of perioperative treatment regimens, 1:2 or 1:1 propensity score-based matching was performed if baseline parameters (age, sex, co-morbidity index) differed between the groups. Different distributions of age, sex, and co-morbidity indices across treatment groups may introduce bias and impact long-term outcomes, so propensity score-based matching was introduced to create well-balanced groups for comparisons of treatment effects. Median overall survival estimates were determined with the Kaplan–Meier method and Cox proportional hazard model. The significance level was set to *p* < 0.05 (two-sided). All confidence intervals (CI) reported are 95% confidence intervals.

## 3. Results

### 3.1. Patient Cohort and Baseline Parameters

**Figure 1:**
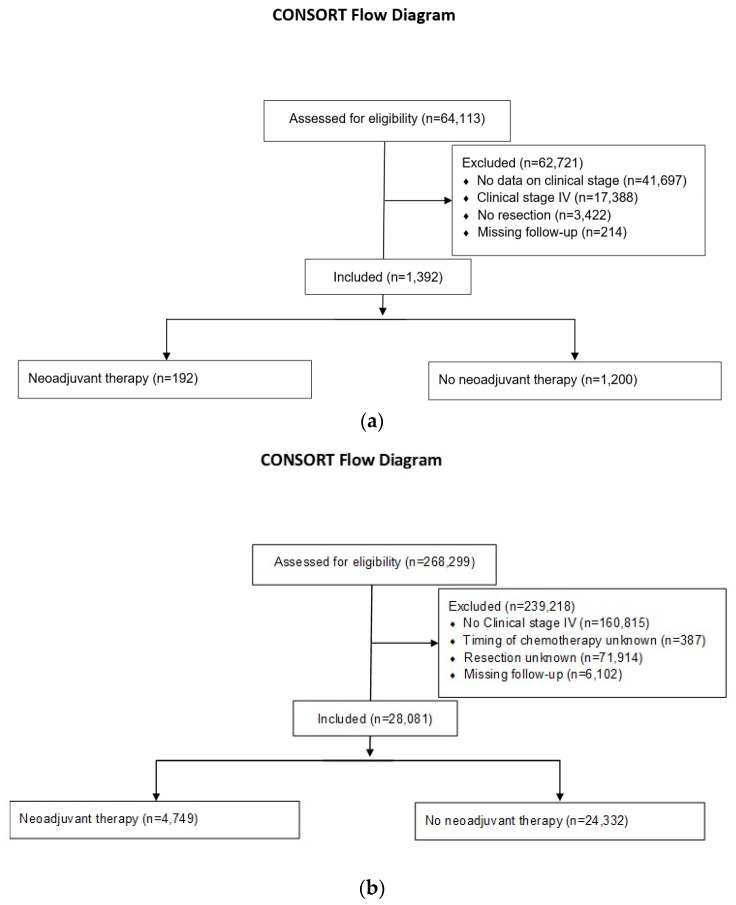
(**a**) Consort 2015 flowchart patient selection GCRG/ADT registry. (**b**) Consort 2015 flowchart patient selection NCDB registry.

A total of 64,113 patients with histologically confirmed PDAC were identified from the GCRG/ADT registry. 62,721 patients were excluded from the study, Figure 1a. A total of 268,299 patients with histologically confirmed PDAC were identified from the NCDB registry.239,218 patients were excluded from the study, Figure 1b.

For patients from the GCRG/ADT registry, the median age was 69 (range 23–89), and 48.6% of the patients were female. A total of 46.9% of the patients had an EGOG performance status of 0, Table 1. Patients were dichotomized into clinical stage IA-IIA versus IIB-III, differentiating patients with and without lymph node involvement. Perioperative regimens stratified for clinical stage are displayed in Table 2.

For patients from the NCDB registry, the median age was 67 (range 21–90), and 49.3% of the patients were female. A total of 66.1% of the patients had a Charlson-Deyo co-morbidity index of 0, Table 3 Perioperative regimens stratified for clinical stage are displayed in Table 4.

Regarding baseline parameters in the GCRG/ADT registry, the respective perioperative therapy patient cohorts were not well balanced. Patients who underwent OP alone and OP + adj patients were older than neo + OP and neo + OP + adj patients (median 69 and 68 years vs. median 62 and 62 years, *p* = 0.010). The cohorts of OP alone patients, neo + OP patients, OP + adj and neo + OP + adj patients, as well as neoRCTX + OP and neoCTX + OP patients, were well-balanced for all other baseline parameters, Supplementary Tables S1 and S2.

Correspondingly, patients who underwent OP alone were older than neo + OP, OP + adj, and neo + OP + adj patients (median 69 years vs. median 64 and 65 and 64 years, *p* = 0.001) in the NCDB registry. Furthermore, surgery alone patients were more likely to have a Charlson-Deyo co-morbidity index of 1 or higher as compared to those patients receiving perioperative therapy (37.0% vs. 33.2%, *p* < 0.001), and there was a disbalance of sex between the perioperative therapy cohorts. neoRCTX + OP patients were younger (median 63 years vs. median 64 years, *p* = 0.018) and there was a trend for higher rates of Charlson-Deyo co-morbidity index of 1 or more (33.3% vs. 30.4%, *p* = 0.068), Supplementary Tables S3 and S4.

### 3.2. Neoadjuvant Therapy and Histopathological Staging

Histopathological parameters were analyzed for patients undergoing neoadjuvant therapy and compared to those with upfront resection. The analysis of the GCRG/ADT registry disclosed a reduction in T as well as N category associated with neoadjuvant therapy, Supplementary Table S5.

The analysis of the NCDB registry demonstrated a similar reduction in T and N stages associated with neoadjuvant therapy. Patients undergoing neoadjuvant therapy were more likely to be diagnosed with negative resection margins as compared to those undergoing upfront surgery (84.7% vs. 77.6%, *p* < 0.001), Supplementary Table S6.

### 3.3. Perioperative Chemotherapy Regimens

Patients who underwent perioperative therapy were stratified as having received neoadjuvant therapy and resection (neo + OP) or resection and adjuvant therapy (OP + adj) or neoadjuvant therapy and resection and adjuvant therapy (neo + OP + adj). Of the 192 patients with neoadjuvant therapy in the GCRG/ADT registry, 126 had neoadjuvant chemotherapy, and 66 had neoadjuvant radiochemotherapy. Both groups contain patients who underwent only neoadjuvant therapy and patients that underwent neoadjuvant and adjuvant therapy. In 126 patients with neoadjuvant chemotherapy, 56 (44.4%) had neoadj + OP and 70 (55.6%) had neoadj + OP + adj. In 66 patients with neoadjuvant radiochemotherapy, 39 (59.1%) had neoadj + OP and 27 (40.9%) had neoadj + OP + adj. Patients identified from the GCRG/ADT registry were more likely to undergo multi-agent than single-agent chemotherapy if receiving neoadjuvant therapy (57.9% vs. 42.1%). While single-agent chemotherapy was performed in the majority of OP + adj patients (67.0%), multi-agent chemotherapy was more often administered to neo + OP + adj patients (67.1%, *p* < 0.001). Patients undergoing neoadjuvant therapy were further stratified as receiving neoadjuvant radiochemotherapy (neoRCTX + OP) or neoadjuvant chemotherapy alone (neoCTX + OP). While neoRCTX + OP patients were more likely to receive single-agent therapy (60.9%), multi-agent therapy was more likely administered to neoCTX + OP patients (66.3%, *p* = 0.024), Table 5.

Patients identified from the NCDB registry were also more likely to undergo multi-agent than single-agent chemotherapy if receiving neoadjuvant therapy (60.5% vs. 39.5%). While single-agent chemotherapy was performed in the majority of OP + adj patients too (68.1%), multi-agent chemotherapy was more often administered to neo + OP + adj patients (84.6%, *p* < 0.001). NeoRCTX + OP patients were more likely to receive multi-agent therapy (52.4%), the rate of multi-agent therapy was higher in neoCTX + OP patients (80.2%, *p* < 0.001), Table 6.

### 3.4. Perioperative Treatment Regimens and Long-Term Outcomes

**Figure 2:**
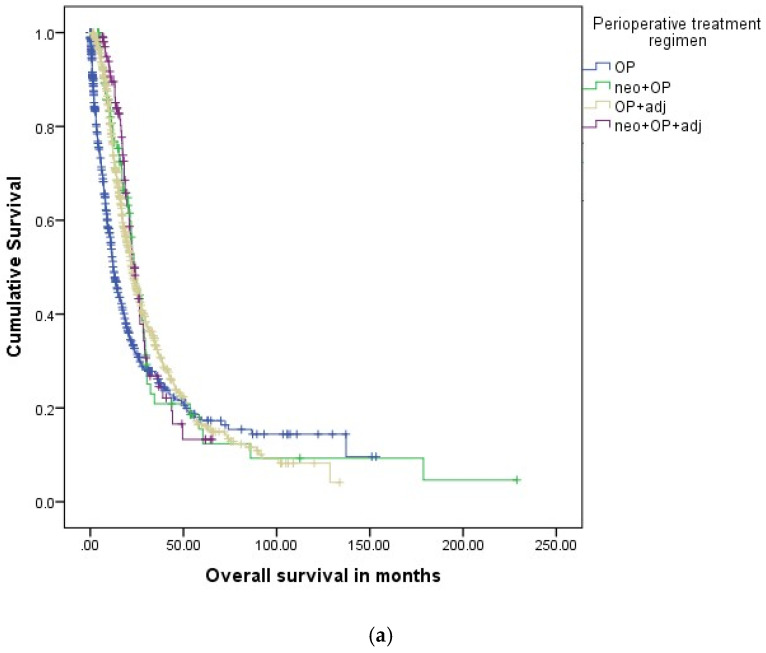

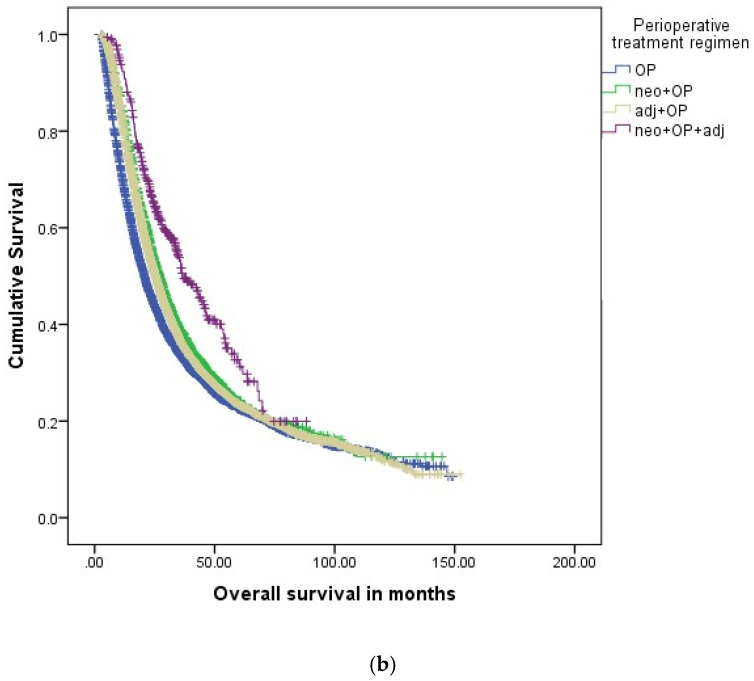
(**a**) Overall survival in perioperative treatment regimens in clinical stage IA-III PDAC (GCRG/ADT registry). (**b**) Overall survival in perioperative treatment regimens in clinical stage IA-III PDAC (NCDB registry). PDAC: pancreatic ductal adenocarcinoma; OP: operation; neoadj.: neoadjuvant; adj.: adjuvant.

In the GCRG/ADT registry, 1:2 propensity score-based matching (neo + OP vs. OP alone and neo + OP vs. OP + adj) was performed prior to head-to-head survival analyses of the perioperative treatment cohorts. OP alone was associated with impaired overall survival (OS) when compared to neo + OP patients (11.3 m vs. 17.8 m, HR 0.820, 95%CI 0.580–0.956, *p* = 0.025), OP + adj patients (11.3 m vs. 18.2 m, HR 0.767, 95%CI 0.598–0.769, *p* = 0.019) and neo + OP + adj patients (11.3 m vs. 21.3 m, HR 0.710, 95%CI 0.511–0.949, *p* = 0.012), Figure 2a and Table 7. For patients with clinical stage IA-IIA, median OS rates were improved for neo + OP patients (13.3 m vs. 23.7 m, HR 0.789, 95%CI 0.370–0.896, *p* = 0.013) as compared to OP alone. For stage IIB-III patients, neo + OP (10.0 m vs. 17.7 m, HR 0.764, 95%CI 0.460–0.843, *p* = 0.041) was superior to OP alone in terms of OS rates. Neo + OP was not associated with improved OS rates as compared to OP + adj for all stages. Median overall survival for neo + OP + adj was 21.3 months as compared to 18.2 months for patients with OP + adj (HR 0.977, 95%CI 0.782–1.113, *p* = 0.071) for all stages. Similarly for stage IA-IIA (neo + OP + adj 24.0 months vs. OP + adj 23.0 months, HR 0.946, 95%CI 0.823–1.342, *p* = 0.121) and stage IIB-III (neo + OP + adj 18.4 months vs. OP + adj 19.9 months, HR 0.824, 95%CI 0.698–1.421, *p* = 0.098) neither neo + OP + adj nor OP + adj were superior in terms of overall survival rates. Patients with neo + OP + adj showed prolonged median overall survival as compared to neo + OP for all patients (17.8 m vs. 21.3 m, HR 0.829, 95%CI 0.622–0.987) and for stage IIB-III patients (17.7 m vs. 19.9 m, HR 0.876, 95%CI 0.516–0.987), Supplementary Figure S1. There was no difference in OS rates for neoRCTX + OP patients as compared to neoCTX + OP patients, Supplementary Table S7.

**Figure 3:**
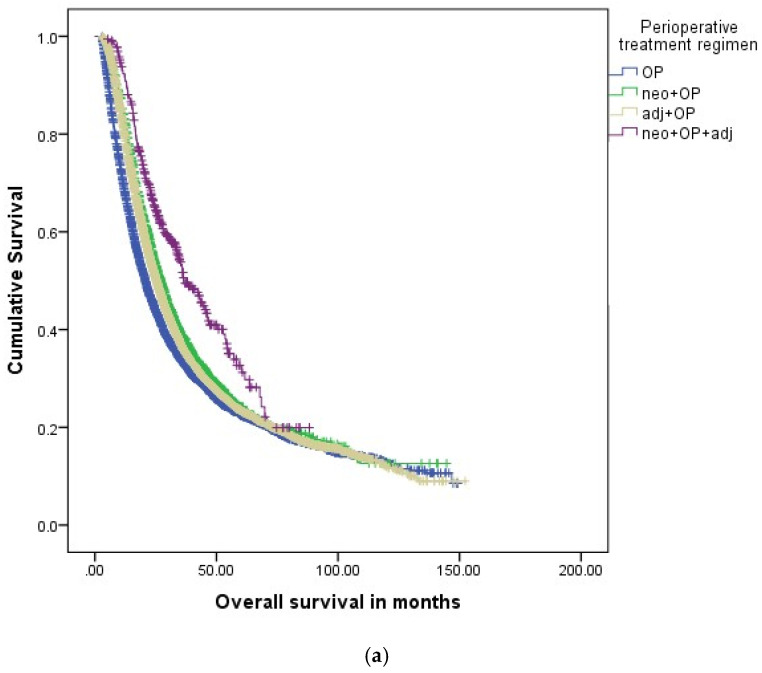

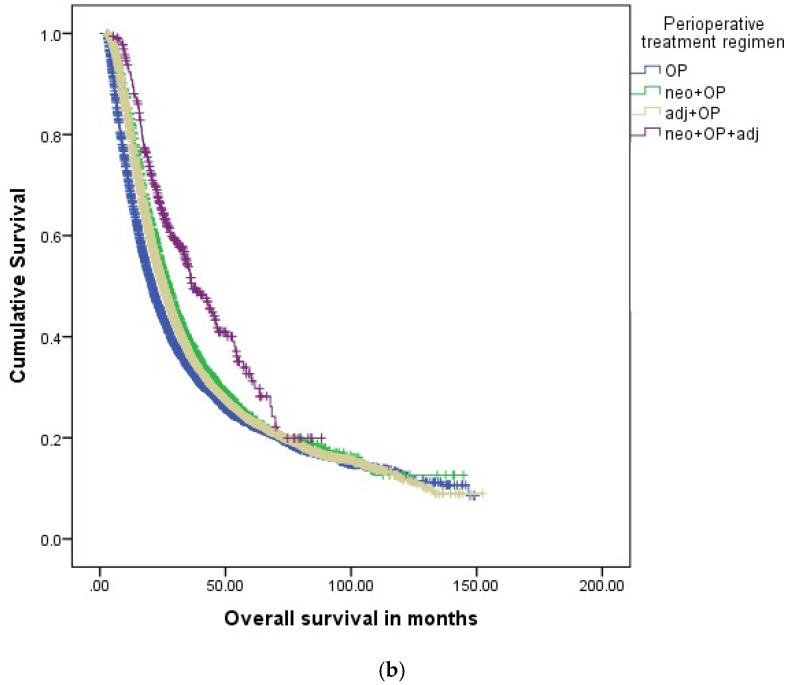
(**a**) Overall survival in perioperative treatment regimens in clinical stage IA-IIA PDAC (NCDB registry). (**b**) Overall survival in perioperative treatment regimens in clinical stage IIB-III PDAC (NCDB registry). OP: operation; neoadj.: neoadjuvant; adj.: adjuvant.

In the NCDB registry, 1:2 propensity score-based matching (neo + OP vs. OP alone and neo + OP vs. OP + adj) and 1:1 propensity score-based matching (neoRCTX + OP vs. neoCTX + OP) was performed prior to head-to-head survival analyses of the perioperative treatment cohorts. Surgery alone was associated with impaired OS as compared to neo + OP (18.3 m vs. 26.4 m, HR 0.730, 95%CI 0.698–0.764, *p* < 0.001), Figure 2b and Table 8. In clinical stage IA-IIA, PDAC neo + OP was associated with improved median OS rates as compared to patients with surgery alone (20.7 m vs. 27.1 m, HR 0.805, 95%CI 0.759–0.853, *p* < 0.001), Figure 3a. Median OS rates were also significantly better for clinical stage IIB-III patients with neo + OP (13.6 m vs. 25.8 m, HR 0.530, 95%CI 0.493–0.570, *p* < 0.001) and neo + OP + adj (13.6 m vs. 28.6 m, HR 0.464, 95%CI 0.388–0.555, *p* < 0.001), Figure 3b. As compared to OP + adj, patients receiving neo + OP showed prolonged median OS rates in both clinical stage IA-IIA (27.1 m vs. 25.3 m, HR 1.066, 95%CI 1.010–1.126, *p* < 0.001) and stage IIB-III (25.8 m vs. 20.8 m, HR 1.305, 95%CI 1.225–1.390, *p* < 0.001), Table 9. Neo + OP + adj was associated with improved OS rates as compared to neo + OP for both stage IA-IIA (27.1 m vs. 36.6 m, HR 0.716, 95%CI 0.614–0.836, *p* < 0.001) and IIB-III patients (25.8 m vs. 28.6 m, HR 0.860, 95%CI 0.717–0.978, *p* < 0.001). There was no difference in OS rates for neoRCTX + OP patients as compared to neoCTX + OP patients.

## 4. Discussion

The cross-validation of perioperative therapy concepts and outcomes comparing the National Cancer Database (NCDB) and the German Cancer Registry Group of the Society of German Tumor Centers—Network for Care, Quality, and Research in Oncology, Berlin (GCRG/ADT) demonstrated that patient selection, and the use of multi-agent concepts in perioperative PDAC therapy remain widely consistent across the registries. Neoadjuvant therapy, when compared to upfront surgery, resulted in improved median overall survival for all clinical stages in both registries, while neoadjuvant radiochemotherapy (neoRCTX + OP) was not superior to neoadjuvant chemotherapy alone (neoCTX + OP). This is the first study to show that neoadjuvant therapy combined with adjuvant therapy (neo + OP + adj) was associated with improved overall survival rates when compared to neoadjuvant (neo + OP) or adjuvant therapy alone (OP + adj) in both stages IA-IIA and IIB-III PDAC after propensity score-based matching.

National cancer registries have been established over the past years to serve as a measure to assure quality control and to evaluate treatment effects and outcomes on a nationwide scale [9,13]. These registries provide major insights into current treatment practice for PDAC, among other cancer entities. Beyond evaluation of clinical practice standards, these registries provide platforms to validate treatment effects observed in clinical trials on “real-world” data with nationwide coverage [9]. The large-scale set-up of national registries makes them ideal databases to address current controversies of perioperative therapy in PDAC to gain insights into treatment outcomes beyond the settings of clinical trials. While national registries are increasingly used to evaluate PDAC treatment effects on a national level [13,14,15], very few international cross-validations of registry studies have been performed so far [8,16].

The German Cancer Registry Group of the Society of German Tumor Centers—Network for Care, Quality, and Research in Oncology, Berlin (GCRG/ADT) is a joint organization of the German Cancer Centers and combines the regional German cancer registries in the national German cancer registry covering all patients treated for any cancer entity in Germany [17,18]. The U.S.-American NCDB is sponsored by the American College of Surgeons and the Commission on Cancer and represents a clinical oncology database sourced from hospital registry data. The NCDB covers 70% of the newly diagnosed cancer cases in the United States [19,20]. These two registries cover the main part of the respective PDAC patient national caseloads and provide large-scale cohorts to compare perioperative treatment concepts and outcomes in patients with clinical stage IA-III PDAC. Patient selection for different perioperative treatment concepts was similar in both registries. Patients selected for neoadjuvant regimens were younger than those receiving adjuvant therapy or surgery alone in both registries. In the NCDB registry, patients receiving OP alone were more likely to have a higher co-morbidity index when compared to patients receiving perioperative treatment. This clinical practice reflects statements by both the U.S.-American National Comprehensive Cancer Network (NCCN) Guideline Pancreatic Adenocarcinoma and the German S3 guideline for the treatment of pancreatic cancer [21,22]. Both national guidelines recommend deescalating or avoiding perioperative therapy in elderly or multi-morbid PDAC patients if reasonable.

Neoadjuvant therapy was performed in 12% of the patients in NCDB as compared to 3% in the GCRG/ADT registry for stages IA-IIA, and 19% as compared to 10% for the stages IIB-III. Moreover, the total numbers of patients in the study with neo + OP or neo + OP + adj for stage IA-III are much lower for the GCRG/ADT registry (*n* = 192) as compared to the NCDB (*n* = 4749). This difference is most likely associated with divergent recommendations in national treatment guidelines. While neoadjuvant therapy was already recommended by the U.S.-American NCCN guideline for PDAC in 2014 in selected patients with borderline resectable PDAC, it is currently only considered an option in high-risk resectable PDAC patients and is encouraged in the context of clinical trials [22,23]. The German S3 guideline for the treatment of pancreatic cancer recommends more restricted use of neoadjuvant therapy [21]. While neoadjuvant therapy may be considered in PDAC patients with locally advanced disease, it is not recommended in resectable patients outside of clinical trials. Thus, neoadjuvant therapy is not routinely performed in Germany, and total numbers of PDAC patients who underwent neoadjuvant therapy entered into the GCRG/ADT registry are lower when compared to the NCDB. Furthermore, adjuvant therapy is recommended for all stages by the German S3-guideline. Therefore, adjuvant therapy, in addition to neoadjuvant therapy, is relatively common in Germany as compared to the U.S. Despite these differences, the use of multi-agent chemotherapy remained similar in both registries. Chemotherapy agents used in perioperative concepts are not available from the NCDB and still incomplete for the GCRG/ADT registry. However, both registries provide data regarding the use of single- and multi-agent chemotherapy. Interestingly, the current study showed improved survival rates with neoadjuvant therapy as compared to adjuvant therapy. In the context of neoadjuvant therapy, multi-agent therapies were the most common strategy in both registries. These combination therapies involve gemcitabine and nab-paclitaxel or FOLFIRINOX, which proved superior to standard single-agent therapies [20]. For adjuvant therapy, the majority of patients in both registries received single-agent therapy. The most common single-agent strategy for PDAC involves gemcitabine alone [21]. Recent studies showed superior long-term outcomes for multi-agent therapies such as gemcitabine and nab-paclitaxel or FOLFIRINOX as compared to single-agent therapies such as gemcitabine alone [7]. Therefore, the higher percentage of patients receiving single-agent therapies in the adjuvant as compared to the neoadjuvant setting might explain improved overall survival rates for patients with neoadjuvant as compared to adjuvant therapy.

In general, overall survival times were shorter for patients from the GCRG/ADT registry as compared to the NCDB; the most considerable difference is found in the group of patients with neoadjuvant and adjuvant therapy (21.3 months versus 35.4 months). There are several potential explanations for this discrepancy. First, the study period for the GCRG-ADT registry started in 2000, while it only started in 2004 for the NCDB. Therefore, it may be hypothesized that the GCRG-ADT registry includes more patients with currently outdated chemotherapy concepts. Furthermore, the first-line chemotherapy agents used in neoadjuvant and adjuvant settings differ in the United States and in Germany. While gemcitabine with or without capecitabine is still recommended by the German S3-guideline, the NCCN started recommending multi-agent therapies such as gemcitabine and nab-paclitaxel, and FOLFIRINOX that proved superior in terms of oncological outcomes [21].

Neoadjuvant therapy has become an important part of PDAC perioperative therapy over the past years [3,5,7,20,24]. The main rationale for neoadjuvant therapy in PDAC is to achieve a downstaging of tumors and to improve resectability [24,25,26]. Our study demonstrated impressive downstaging of tumors in terms of T and N stage for patients undergoing neoadjuvant therapy across registries. Despite successful shrinkage of tumors, tumor burden was not eliminated by neoadjuvant therapy in the vast majority of patients. Recent consensus guidelines suggest focusing on the remaining tumor burden as an important parameter to determine the efficacy of neoadjuvant therapy [27]. While growing evidence supports the benefits of neoadjuvant therapy, the ideal sequence and treatment regimen of perioperative therapy has yet to be determined [7,28]. It is unclear whether neoadjuvant therapy is superior to adjuvant therapy or if a combination of neoadjuvant and adjuvant therapy is more beneficial than each of these options alone. This study is the first to assess the sequence of perioperative therapy of stage IA-III PDAC patients. Neoadjuvant therapy was associated with prolonged overall survival as compared to adjuvant therapy for both clinical stage IA-IIA and IIB-III PDAC in the NCDB registry (27.1 m vs. 25.3 m and 25.8 m vs. 20.8 m, respectively). These results were not confirmed in the GCRG/ADT, and overall survival was similar for patients with neoadjuvant and adjuvant therapy across all stages. The combination of neoadjuvant and adjuvant therapy was associated with considerably higher overall survival rates than either perioperative treatment alone for clinical stage IA-IIA (36.6 m) and IIB-III (28.6 m). These results were confirmed in the GCRG/ADT registry showing prolonged overall survival rates for all patients and for patients with stage IIB-III but not stage IA-IIA. It may be speculated that the analysis was underpowered due to low absolute patient numbers in the groups of neoadjuvant therapy alone and even more so for combined neoadjuvant and adjuvant therapy. Very few other studies have addressed the issue of combining neoadjuvant and adjuvant therapy in PDAC. Watson et al. performed an analysis of clinical stage 0-II PDAC patients from the NCDB and found improved overall survival with neoadjuvant and adjuvant therapy as compared to neoadjuvant therapy alone [28]. Drake et al. found that additional adjuvant therapy is particularly beneficial in patients with microscopically incomplete R1 resection [29].

While neoadjuvant therapy was associated with prolonged overall survival, this study failed to demonstrate the benefit of neoadjuvant radiochemotherapy over neoadjuvant chemotherapy alone for both registries. Radiotherapy is often integrated into standard neoadjuvant concepts in the United States and is recommended by the current NCCN guideline [22]. In Germany, however, the use of radiotherapy in the context of neoadjuvant or adjuvant therapy for PDAC is discouraged by the German S3 guideline for the treatment of pancreatic cancer [21]. While there is evidence supporting neoadjuvant chemotherapy with and without radiochemotherapy, respectively, head-to-head comparisons of neoadjuvant radiochemotherapy versus neoadjuvant chemotherapy alone are rare [7]. Trinh et al. assessed neoadjuvant chemotherapy alone versus neoadjuvant chemoradiation for resectable and borderline resectable PDAC and found no difference in overall survival [30]. An analysis of the NCDB for the time period from 2004 to 2013 also failed to show a difference in overall survival for resected PDAC with neoadjuvant radiochemotherapy versus neoadjuvant chemotherapy alone [25].

While perioperative therapy primarily consists of classical single- or multi-agent chemotherapy regimens, a growing body of evidence suggests that PDAC tumors are heterogeneous and more individualized approaches are warranted [31,32,33,34]. Multiple PDAC therapeutic targets have been identified, including mismatch repair deficiency, microsatellite instability, or BRCA mutations [31,32]. Immunotherapy might help to improve long-term outcomes in these and other patient subgroups [27]. Future perioperative therapy should take molecular tumor characteristics into account and involve not only multi-agent chemotherapies but also combined immunotherapy.

This study has several limitations. Details about chemotherapy agents were not available from the NCDB and only partially available from the GCRG/ADT registry. Molecular marker profiles or mutational analyses could also not be derived from the registries. Therefore, detailed analysis of perioperative therapy regimens and distinct agents and individualized therapy could not be performed. A further limitation is a change in national treatment guidelines over the course of the study period, leading to the inclusion of patients with different perioperative therapy regimens and indications. The national registries are derived from population-based retrospective databases, and inaccuracy in data collection cannot be ruled out. Furthermore, patients with missing data had to be excluded introducing potential selection bias that is difficult to account for at the scale of this study. However, the national coverage of PDAC cases is about 70% for the NCDB and 100% for the GCRG/ADT registry. Therefore, these databases provide two of the largest national PDAC cohorts to study perioperative therapy on “real-world data”.

## 5. Conclusions

In conclusion, we present the first cross-validation study of the NCDB and GCRG/ADT registries assessing perioperative treatment concepts and outcomes in PDAC. While neoadjuvant therapy is still not routinely performed in Germany as compared to the U.S., patient selection and treatment modalities are similar in both registries. Neoadjuvant therapy combined with adjuvant therapy was associated with improved prognosis as compared to neoadjuvant therapy alone.

## Figures and Tables

**Table 1:** Baseline parameters and clinical staging GCRG/ADT registry.

**Parameter**		**Baseline Parameters**	
		***n***	**%/Range**
***n***	**Condition**	1392	
**Age**		69	23–89
**Sex**	Male	715	51.4%
	Female	677	48.6%
**ECOG**	0	654	46.9%
	1	611	43.8%
	2	125	9.1%
	3	1	0.1%
	4	1	0.1%
**Parameter**		**Clinical Stage**	
		*n*	%
***n***		1392	
**UICC Stage**			
**Stage IA**		114	8.2%
**Stage IB**		319	22.9%
**Stage IIA**		262	18.8%
**Stage IIB**		469	33.7%
**Stage III**		228	16.4%

**Table 2:** Clinical stages and perioperative therapy GCRG/ADT registry.

**Parameter**	**Stage IA-IIA**	
	***n***	**%**
***n***	695	
**Treatment Regimen**		
**OP alone**	290	41.7%
**Neoadj. therapy + OP**	24	3.5%
**OP + adj. therapy**	359	51.7%
**Neoadj. therapy + OP + adj. therapy**	22	3.2%
**Parameter**	**Stage IIB-III**	
	***n***	**%**
***n***	697	
**Treatment Regimen**		
**OP alone**	219	31.4%
**Neoadj. therapy + OP**	71	10.2%
**OP + adj. therapy**	332	47.6%
**Neoadj. therapy + OP + adj. therapy**	75	10.8%

OP: operation; neoadj.: neoadjuvant; adj.: adjuvant.

**Table 3:** Baseline parameters and clinical staging NCDB registry.

**Parameter**		**Baseline Parameters**	
		***n***	**%/Range**
***n***	**Condition**	29,081	
**Age**		67	21–90
**Sex**	Male	14,757	50.7%
	Female	14,324	49.3%
**Charlson-Deyo Co-morbidity Score**	0	19,210	66.1%
	1	7861	27.0%
	2 or higher	2010	6.9%
**Parameter**		**Clinical Stage**	
		*n*	%
***n***		29,081	
**UICC Stage**			
**Stage IA**		4080	14.0%
**Stage IB**		8212	28.2%
**Stage IIA**		7406	25.5%
**Stage IIB**		7971	27.4%
**Stage III**		1412	4.9%

**Table 4:** Clinical stages and perioperative therapy NCDB registry.

**Parameter**	**Stage IA-IIA**	
	***n***	**%**
***n***	19,698	
**Treatment Regimen**		
**OP alone**	5590	28.4%
**Neoadj. therapy + OP**	2373	12.0%
**OP + adj. therapy**	11,376	57.8%
**Neoadj. therapy + OP + adj. therapy**	359	1.8%
**Parameter**	**Stage IIB-III**	
	***n***	**%**
***n***	9383	
**Treatment Regimen**		
**OP alone**	2077	22.1%
**Neoadj. therapy + OP**	1799	19.2%
**OP + adj. therapy**	5289	56.4%
**Neoadj. therapy + OP + adj. therapy**	218	2.3%

OP: operation; neoadj.: neoadjuvant; adj.: adjuvant.

**Table 5:** Perioperative Therapy and Chemotherapy Regimens GCRG/ADT Registry.

**Parameter**	**Condition**	**Total**		**Neoadj. Therapy + OP**		**OP + Adjuvant Therapy**		**Neoadj. Therapy + OP + Adjuvant Therapy**		***p***
		***n***	**%**	***n***	**%**	***n***	**%**	***n***	**%**	
*n*		883		95		691		97		-
**Single-agent Chemotherapy**		539	61.0%	40	42.1%	463	67.0%	32	32.9%	
**Multi-agent Chemotherapy**		344	39.0%	55	57.9%	228	33.0%	65	67.1%	**0.001**
**Parameter**		**Neoadj. Chemotherapy + OP**		**Neoadj. Radiochemotherapy + OP**						
	**Condition**	*n*	%	*n*	%	*p*				
*n*		126		66						
**Single-agent Chemotherapy**		45	35.7%	40	60.6%					
**Multi-agent Chemotherapy**		81	64.3%	26	39.4%	**0.026**				

OP: operation; neoadj.: neoadjuvant; adj.: adjuvant.

**Table 6:** Perioperative Therapy and Chemotherapy Regimens NCDB Registry.

**Parameter**	**Condition**	**Total**		**Neoadj. Therapy + OP**		**OP + Adjuvant Therapy**		**Neoadj. Therapy + OP + Adjuvant Therapy**		***p***
		***n***	**%**	***n***	**%**	***n***	**%**	***n***	**%**	
***n***		19,740		3887		15,283		570		-
**Single-agent Chemotherapy**		12,029	60.9%	1534	39.5%	10,407	68.1%	88	15.4%	
**Multi-agent Chemotherapy**		7711	39.1%	2353	60.5%	4876	31.9%	482	84.6%	**0.001**
**Parameter**		**Neoadj. Chemotherapy + OP**		**Neoadj. Radiochemotherapy + OP**						
	**Condition**	*n*	%	*n*	%	*p*				
***n***		1137		2750						
**Single-agent Chemotherapy**		225	19.8%	1309	47.6%					
**Multi-agent Chemotherapy**		912	80.2%	1441	52.4%	**0.001**				

OP: operation; neoadj.: neoadjuvant; adj.: adjuvant.

**Table 7:** Perioperative treatment cohorts and overall survival GCRG/ADT registry.

**All Patients**				
**Parameter**	**Median Survival (Months)**	**HR**	**95%CI**	***p* Univariate**
**OP alone**	11.3			
**Neoadj. therapy + OP**	17.8	0.820	0.580–0.956	**0.025**
**OP + adj. therapy**	18.2	0.767	0.598–0.769	**0.019**
**Neoadj. therapy + OP + adj. therapy**	21.3	0.710	0.511–0.949	**0.012**
**Stage IA-IIA**				
**Parameter**	**Median Survival (Months)**	**HR**	**95%CI**	***p* Univariate**
**OP alone**	13.3			
**Neoadj. therapy + OP**	23.7	0.789	0.370–0.896	**0.013**
**OP + adj. therapy**	23.0	0.749	0.537–0.802	**0.003**
**Neoadj. therapy + OP + adj. therapy**	24.0	0.969	0.570–0.946	**0.049**
**Stage IIB-III**				
**Parameter**	**Median Survival (Months)**	**HR**	**95%CI**	***p* Univariate**
**OP alone**	10.0			
**Neoadj. therapy + OP**	17.7	0.764	0.460–0.843	**0.041**
**OP + adj. therapy**	18.4	0.498	0.397–0.742	**0.003**
**Neoadj. therapy + OP + adj. therapy**	19.9	0.498	0.355–0.797	**0.011**

OP: operation; neoadj.: neoadjuvant; adj.: adjuvant.

**Table 8:** Perioperative treatment cohorts and overall survival NCDB registry.

**All Patients**				
**Parameter**	**Median Survival (Months)**	**HR**	**95%CI**	***p* Univariate**
**OP alone**	18.3			
**Neoadj. therapy + OP**	26.4	0.730	0.698–0.764	**<0.001**
**OP + adj. therapy**	23.6	0.817	0.792–0.843	**<0.001**
**Neoadj. therapy + OP + adj. therapy**	35.4	0.562	0.501–0.630	**<0.001**
**Stage IA-IIA**				
**Parameter**	**Median Survival (Months)**	**HR**	**95%CI**	***p* Univariate**
**OP alone**	20.7			
**Neoadj. therapy + OP**	27.1	0.805	0.759–0.853	**<0.001**
**OP + adj. therapy**	25.3	0.856	0.824–0.889	**<0.001**
**Neoadj. therapy + OP + adj. therapy**	36.6	0.579	0.499–0.672	**<0.001**
**Stage IIB-III**				
**Parameter**	**Median Survival (Months)**	**HR**	**95%CI**	***p* Univariate**
**OP alone**	13.6			
**Neoadj. therapy + OP**	25.8	0.530	0.493–0.570	**<0.001**
**OP + adj. therapy**	20.8	0.672	0.636–0.711	**<0.001**
**Neoadj. therapy + OP + adj. therapy**	28.6	0.464	0.388–0.555	**<0.001**

OP: operation; neoadj.: neoadjuvant; adj.: adjuvant.

**Table 9:** Perioperative treatment cohorts and overall survival NCDB registry.

**All Patients**					
Parameter	**Condition**	**Median Survival (Months)**	**HR**	**95% CI**	***p* Univariate**
**Neoadj. Therapy**	Neoadj. therapy	26.4	1.305	1.225–1.390	**<0.001**
	Adj. therapy	23.6			
**Adjuvant Therapy**	Neoadj. therapy and OP	26.4			**<0.001**
	Neoadj. therapy and OP and adj. therapy	35.4	0.860	0.717–1.031	
**Radiochemotherapy**	Neoadj. chemotherapy	26.2			0.730
	Neoadj. radiochemotherapy	26.3	0.730	0.924–1.119	
**Stage IA-IIA**					
**Parameter**	**Condition**	**Median Survival (Months)**	**HR**	**95% CI**	***p* Univariate**
**Neoadj. Therapy**	Neoadj. therapy	27.1	1.066	1.010–1.126	**0.020**
	Adj. therapy	25.3			
**Adjuvant Therapy**	Neoadj. therapy and OP	27.1			**<0.001**
	Neoadj. therapy and OP and adj. therapy	36.6	0.716	0.614–0.836	
**Radiochemotherapy**	Neoadj. chemotherapy	27.1			0.392
	Neoadj. radiochemotherapy	26.3	1.056	0.932–1.196	
**Stage IIB-III**					
**Parameter**	**Condition**	**Median Survival (Months)**	**HR**	**95% CI**	***p* Univariate**
**Neoadj. Therapy**	Neoadj. therapy	25.8	1.305	1.225–1.390	**<0.001**
	Adj. therapy	20.8			
**Adjuvant Therapy**	Neoadj. therapy and OP	25.8			**0.013**
	Neoadj. therapy and OP and adj. therapy	28.6	0.860	0.717–0.978	
**Radiochemotherapy**	Neoadj. chemotherapy	25.1			0.596
	Neoadj. radiochemotherapy	26.1	0.960	0.827–1.115	

OP: operation; neoadj.: neoadjuvant; adj.: adjuvant.

## Data Availability

Complete data are available upon request from the corresponding author.

## Supplementary Materials

The following supporting information can be downloaded at: https://www.mdpi.com/article/10.3390/cancers14040868/s1, Table S1. Baseline Parameters and Perioperative Therapy Cohorts GCRG/ADT registry; Table S2. Baseline Parameters and Neoadjuvant Therapy Subgroups GCRG/ADT registry; Table S3. Baseline Parameters and Perioperative Therapy Cohorts NCDB registry; Table S4. Baseline Parameters and Neoadjuvant Therapy Subgroups NCDB registry; Table S5. Neoadjuvant Therapy and Histopathological Parameters GCRG/ADT Registry; Table S6. Neoadjuvant Therapy and Histopathological Parameters NCDB; Table S7. Perioperative Therapy Cohorts and Overall Survival GCRG/ADT registry; Figure S1. (a) Overall Survival in Perioperative Treatment Regimens in clinical stage IA-IIA PDAC (GCRG/ADT Registry), (b) Overall Survival in Perioperative Treatment Regimens in clinical stage IIB-III PDAC (GCRG/ADT Registry).
