# Supplementary material for: Concepts and Outcomes of Perioperative Therapy in Stage IA-III Pancreatic Cancer—A Cross-Validation of the National Cancer Database (NCDB) and the German Cancer Registry Group of the Society of German Tumor Centers (GCRG/ADT)
Source: Cancers (Basel). 2022 Feb 9;14(4):868. doi: 10.3390/cancers14040868 (PMC8870242; doi:10.3390/cancers14040868)
Supplement: Supplementary file 1 [file cancers-14-00868-s001.zip › cancers-1568959-supplementary.pdf]

Table S1. Baseline Parameters and Perioperative Therapy Cohorts GCRG/ADT registry.

Parameter	Condition	Total		OP Upfront		neoadj. Therapy + OP		OP + Adjuvant Therapy		neoadj. Therapy + OP + Adjuvant Therapy		p
		n or Median	%/Range/SE	n or Median	%/Range/SE	n or Median	%/Range/SE	n or Median	%/Range/SE	n or Median	%/Range/SE	
n		1611		644 (40.0%)		100 (6.2%)		765 (47.5%)		102 (6.3%)		-
**Baseline Parameters**												
**Age**		69	23-86	69	0.42	63	0.91	67	0.32	62	0.92%	**0.002**
**Sex**	male	835	51.8%	325	50.5%	51	51.0%	412	53.9%	47	46.1%	
	female	776	48.2%	319	49.5%	49	49.0%	353	46.1%	55	53.9%	0.374
**ECOG**	0-1	854	53.0%	415	64.4%	49	49.0%	342	44.7%	48	47.4%	
	2-4	757	47.0%	229	35.6%	51	51.0%	423	55.3%	54	52.6%	0.198
**Clinical**	Stage IA	129	8.0%	64	10%	0	0.0%	63	8.2%	2	2.0%	**0.001**
**Staging**	Stage IB	334	20.7%	144	22%	4	4.0%	175	22.9%	11	10.8%	
**UICC**	Stage IIA	310	19.2%	136	21%	20	20.0%	144	18.8%	10	9.8%	
	Stage IIB	594	36.9%	238	37%	20	20.0%	309	40.4%	27	26.5%	
	Stage III	244	15.1%	62	10%	56	56%	74	9.7%	52	51.0%	

OP: operation; neoadj.: neoadjuvant; adj.: adjuvant.

Table S2. Baseline Parameters and Neoadjuvant Therapy Subgroups GCRG/ADT registry.

Parameter		neoadj. Chemotherapy + OP		neoadj. Radiochemotherapy + OP		
		n/Median	%/Range/SE	n/Median	%/Range/SE	p
**n**	**Condition**	134		68		
**Age**		63	0.82	62	0.81	0.876
**Sex**	male	56	44.0%	39	47.4%	
	female	75	46.0%	29	42.6%	0.277
**ECOG**	0-1	62	46.5%	29	42.9%	
	2-3	72	53.5%	39	47.1%	0.389
**Clinical**	Stage IA	2	1.5%	0	0.0%	0.441
**Staging**	Stage IB	10	7.5%	5	7.4%	
**UICC**	Stage IIA	22	16.4%	8	11.8%	
	Stage IIB	34	25.4%	13	19.1%	
	Stage III	66	49.3%	42	61.8%	

OP: operation; neoadj.: neoadjuvant.

Table S3. Baseline Parameters and Perioperative Therapy Cohorts NCDB registry.

Parameter	Condition	Total		OP Upfront		neoadj. Therapy + OP		OP + Adjuvant Therapy		neoadj. Therapy + OP + Adjuvant Therapy		p
		n	%	n	%	n	%	n	%	n	%	
**n**		29081		7667 (26.4%)		4172 (14.3%)		16665 (47.3%)		577 (2.0%)		-
**Baseline Parameters**												
**Age**		67	21-90	69	0.12	64	0.15	65	0.08	64	0.4	**0.001**
**Sex**	male	14757	50.70%	3745	48.80%	2093	50.20%	8601	51.60%	318	55.10%	
	female	14324	49.30%	3922	51.20%	2079	49.80%	8064	48.40%	259	44.90%	**0.001**
**Charlson-Deyo**	0	19210	66.10%	4828	63.00%	2819	67.60%	11158	67.00%	404	70.00%	
**Comorbidity Score**	1 or higher	9871	33.90%	2839	37.00%	1353	32.40%	5506	33.00%	173	30.00%	**0.001**
**Clinical**	Stage IA	4080	14.0%	1361	17.8%	226	5.5%	2456	14.7%	37	6.4%	**0.001**
**Staging**	Stage IB	8212	28.2%	2360	30.8%	738	17.7%	4976	29.9%	139	23.9%	
**UICC**	Stage IIA	7406	25.5%	1869	24.4%	1409	33.8%	3944	23.7%	184	31.9%	
	Stage IIB	7971	27.4%	1923	25.1%	1039	24.9%	4844	29.2%	165	28.6%	
	Stage III	1412	4.9%	154	2.1%	760	18.2%	445	2.5%	53	9.2%	

OP: operation; neoadj.: neoadjuvant; adj.: adjuvant.

Table S4. Baseline Parameters and Neoadjuvant Therapy Subgroups NCDB registry.

Parameter		neoadj. Chemotherapy + OP		neoadj. Radiochemotherapy + OP		
		n	%	n	%	p
**n**	**Condition**	1285		2887		
**Age**		64	0.29	63	0.18	**0.018**
**Sex**	male	636	49.50%	1457	50.50%	
	female	649	50.50%	1430	49.50%	0.569
**Charlson-Deyo**	0	894	69.60%	1925	66.70%	
**Comorbidity Score**	1 or higher	391	30.40%	962	33.30%	**0.068**
**Clinical**	Stage IA	86	8.7%	140	4.9%	0.001
**Staging**	Stage IB	261	20.3%	477	16.5%	
**UICC**	Stage IIA	422	32.8%	987	34.2%	
	Stage IIB	335	26.1%	704	24.4%	
	Stage III	181	14.1%	579	20.1%	

OP: operation; neoadj.: neoadjuvant.

Table S5. Neoadjuvant Therapy and Histopathological Parameters GCRG/ADT Registry.

	Condition	Total		OP Upfront		neoadj. Therapy + OP		p
		n	%	n	%	n	%	
**n**		1392		1200 (86.2%)		192 (13.8%)		**-**
				**Histopathology**				
**pT stage**	T0	17	1.2%	2	0.2%	16	8.4%	
	T1	62	4.5%	49	4.1%	15	7.8%	
	T2	294	21.1%	251	20.9%	44	22.8%	
	T3	966	69.4%	861	71.8%	101	52.7%	
	T4	53	3.8%	37	3.1%	16	8.4%	**<0.001**
**pN stage**	N0	508	36.5%	414	34.5%	94	49.2%	
	N1	785	56.4%	698	58.2%	86	44.7%	
	N2	99	7.1%	88	7.3%	12	6.1%	**0.001**
**R Stage**	R0	988	71.0%	851	70.9%	138	71.9%	
	R+	404	29.0%	349	29.1%	54	28.1%	0.859

Table S6. Neoadjuvant Therapy and Histopathological Parameters NCDB.

Parameter	Condition	Total		OP Upfront		neoadj. Therapy + OP		p
		n or Median	%/Range/SE	n or Median	%/Range/SE	n or Median	%/Range/SE	
**n**		29081		24332		4749		**-**
				**Histopathology**				
**T stage**	T0	2174	7.5%	1460	6.0%	712	15.0%	
	T1	1913	6.6%	1338	5.5%	565	11.9%	
	T2	3951	13.6%	3382	13.9%	570	12.0%	
	T3	20300	69.8%	17568	72.2%	2745	57.8%	
	T4	743	2.6%	584	2.4%	157	3.3%	**<0.001**
**N stage**	N0	12098	41.6%	9149	37.6%	2944	62.0%	**<0.001**
	N+	16983	58.4%	15183	62.4%	1805	38.0%	
**R Stage**	R0	22916	78.8%	18882	77.6%	4022	84.7%	
	R+	6165	21.2%	5450	22.4%	727	15.3%	**<0.001**

OP: operation; neoadj.: neoadjuvant.

Table S7. Perioperative Therapy Cohorts and Overall Survival GCRG/ADT registry.

**All patients**					
**Parameter**	**Condition**	**Median Survival (Months)**	**HR**	**95%CI**	**p Univariate**
**Neoadj. Therapy**	neoadj. Therapy	17.8			
	adj. Therapy	18.2	1.142	0.773-1.410	0.729
**Adjuvant therapy**	neoadj. Therapy and OP	17.8			**0.048**

	Neoadj. Therapy and OP and adj. Therapy	21.3	0.829	0.622-0.987	
**Radiochemotherapy**	Neoadj. Chemotherapy	18.3			
	Neoadj. Radiochemotherapy	17.8	1.125	0.837-1.595	0.702
**Stage IA-IIA**					
**Parameter**	**Condition**	**Median survival (months)**	**HR**	**95%CI**	**p univariate**
**Neoadj. Therapy**	neoadj. Therapy	23.7			
	adj. Therapy	23.0	1.219	0.719-2.398	0.519
**Adjuvant therapy**	neoadj. Therapy and OP	23.7			
	Neoadj. Therapy and OP and adj. Therapy	24.0	1.319	0.649-2.198	0.349
**Radiochemotherapy**	Neoadj. Chemotherapy	20.9			
	Neoadj. Radiochemotherapy	24.2	0.329	0.198-1.198	0.129
**Stage IIB-III**					
**Parameter**	**Condition**	**Median survival (months)**	**HR**	**95%CI**	**p univariate**
**Neoadj. Therapy**	neoadj. Therapy	17.7			
	adj. Therapy	18.4	1.139	0.759-1.398	0.748
**Adjuvant therapy**	neoadj. Therapy and OP	17.7			
	Neoadj. Therapy and OP and adj. Therapy	19.9	0.876	0.516-0.987	**0.048**
**Radiochemotherapy**	Neoadj. Chemotherapy	15.8			
	Neoadj. Radiochemotherapy	17.1	1.423	0.987-2.139	0.149

OP: operation; neoadj.: neoadjuvant; adj.: adjuvant.

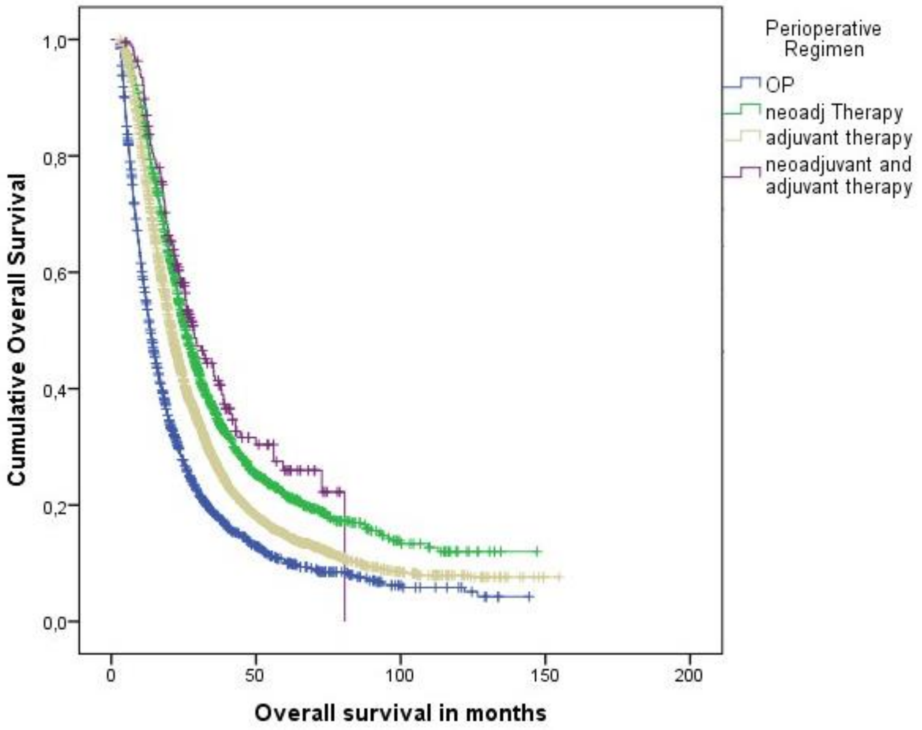

(**a**)

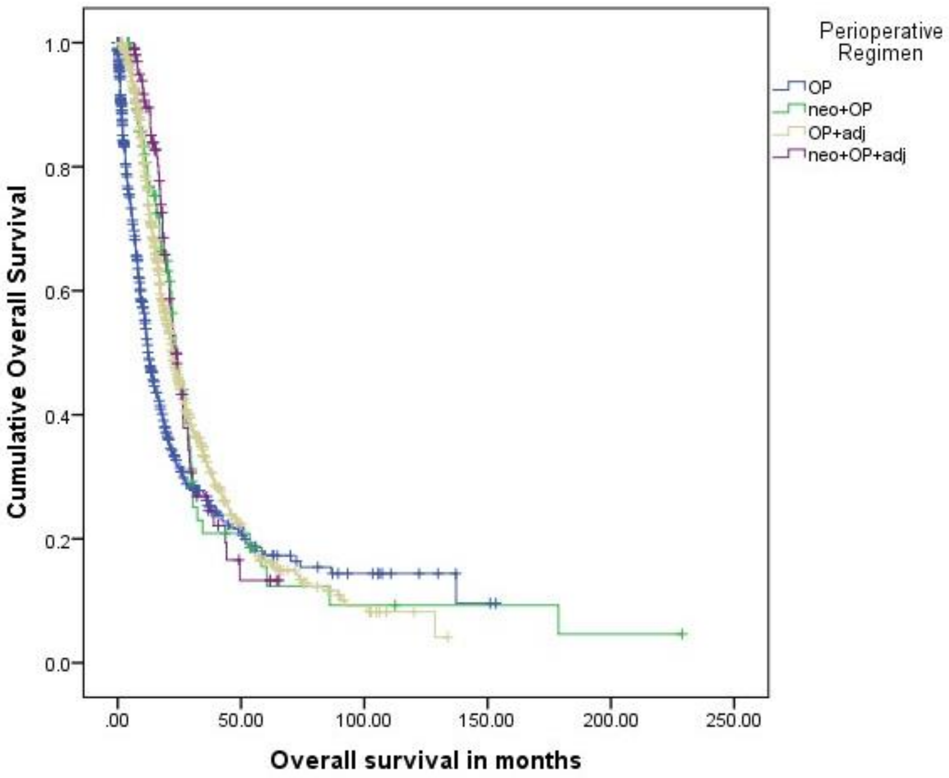

(**b**)

Figure S1. (**a**) Overall Survival in Perioperative Treatment Regimens in clinical stage IA-IIA PDAC (GCRG/ADT Registry), (**b**) Overall Survival in Perioperative Treatment Regimens in clinical stage IIB-III PDAC (GCRG/ADT Registry). OP: operation; neoadj.: neoadjuvant; adj.: adjuvant.
